# Neurobiology Underlying Fibromyalgia Symptoms

**DOI:** 10.1155/2012/585419

**Published:** 2011-10-27

**Authors:** Marta Ceko, M. Catherine Bushnell, Richard H. Gracely

**Affiliations:** ^1^Alan Edwards Centre for Research on Pain, McGill University, 3640 University Street, Room M19, Montreal, QC, H2A 1C1, Canada; ^2^Department of Neurology & Neurosurgery, McGill University, 3640 University Street, Room M19, Montreal, QC, H2A 1C1, Canada; ^3^Department of Anesthesia, McGill University, 3640 University Street, Room M19, Montreal, QC, H2A 1C1, Canada; ^4^Center for Neurosensory Disorders, University of North Carolina, CB No. 7280, 3330 Thurston Building, Chapel Hill, NC 27599, USA

## Abstract

Fibromyalgia is characterized by chronic widespread pain, clinical symptoms that include cognitive and sleep disturbances, and other abnormalities such as increased sensitivity to painful stimuli, increased sensitivity to multiple sensory modalities, and altered pain modulatory mechanisms. Here we relate experimental findings of fibromyalgia symptoms to anatomical and functional brain changes. Neuroimaging studies show augmented sensory processing in pain-related areas, which, together with gray matter decreases and neurochemical abnormalities in areas related to pain modulation, supports the psychophysical evidence of altered pain perception and inhibition. Gray matter decreases in areas related to emotional decision making and working memory suggest that cognitive disturbances could be related to brain alterations. Altered levels of neurotransmitters involved in sleep regulation link disordered sleep to neurochemical abnormalities. Thus, current evidence supports the view that at least some fibromyalgia symptoms are associated with brain dysfunctions or alterations, giving the long-held “it is all in your head” view of the disorder a new meaning.

## 1. Introduction

In order to examine the neurobiology underlying the symptoms of fibromyalgia, we must first determine what those symptoms are. Until recently, fibromyalgia (FM) was diagnosed based on the ARC1990 criteria [1], which were widespread pain in combination with tenderness at 11 or more of 18 specific tender point sites. The provisional ACR 2010 FM diagnostic criteria [2], suggested as an alternative method of diagnosing FM, do not require the presence of tenderness, but rather include a list of several other symptoms, including fatigue, unrefreshing sleep, and cognitive symptoms, as well as a mix of some other symptoms that could include headache, depression, and lower abdominal pain/cramping. The hallmark symptom is still widespread pain, and a diagnosis of fibromyalgia requires this symptom. However, a patient must also have some of the other symptoms that are common among FM patients in order to reach a composite score that would lead to a diagnosis of FM. In addition to clinical symptoms that make up the diagnosis of FM, experimental studies have identified a number of other abnormalities in FM patients, including increased sensitivity to multiple types of painful stimuli, increased sensitivity to other sensory modalities, and alterations in pain modulatory mechanisms. Further, neuroimaging studies have found functional, anatomical, and neurochemical differences in the brains of FM patients compared to healthy control subjects. Most of the clinical symptoms associated with FM have not been systematically studied in the experimental setting, but there are a number of studies that have provided an objective evaluation of the altered cognitive functioning and sleep disturbances reported in FM patients. Thus, this paper will focus on the experimental evidence related to FM symptoms and connect these perceptual and cognitive signs to abnormalities observed in the brains of FM patients.

### 1.1. Altered Pain Perception in FM Patients

The hallmark symptom of FM is widespread ongoing musculoskeletal pain. In addition, FM patients have been distinguished from other patients with widespread pain syndromes primarily by the presence of tenderness that has been assessed clinically by finding pain evoked by 4 kg manual pressure in at least 11 of 18 defined tender points. This tender point concept was not based on an understanding of the underlying pathophysiology, but rather on empirical observation. Thus, although the ARC-90 diagnostic criteria provided an important uniform tool for defining the FM syndrome, they did not validate the tender point concept, due to the circular evidence on which the criteria were based [3]. In fact, much evidence indicates that tender points are just sites normally more sensitive to pressure pain in all individuals [4–7] and that FM patients have an increased pressure sensitivity at non-tender-point sites as well [8]. Accumulating evidence now shows that FM patients have increased sensitivity to many types of painful stimulation, including pressure at non-tender-point sites [9], heat and cold pain [6, 10–14], electrical stimulation [6], and intramuscular hypertonic saline injection [15]. Despite the plethora of evidence for hypersensitivity to painful stimuli, there is less evidence that FM patients are more sensitive to innocuous somatosensory stimuli. Detection thresholds for tactile and electrical stimuli are not altered in FM [6, 12, 13], but Hollins et al. [16] found that FM patients rated innocuous pressure as more intense than did healthy controls, although the effects in the innocuous range were weaker than in the noxious range. The evidence for changes in cool or warm detection also is mixed, with most investigators finding no differences between FM and controls for heat [6, 10] or cold [10, 12], whereas one study found FM patients to have reduced heat detection thresholds [12], and one study found patients to have reduced cold detection thresholds [6]. Thus, it appears that the altered sensitivity within the somatosensory system is more profound in the noxious range than in the innocuous range.

### 1.2. Evidence for Generalized Hypersensitivity to Unpleasant Stimuli

The hypersensitivity of FM patients to painful stimuli has led some investigators to propose that fibromyalgia involves a hypervigilance to pain and pain-associated information [17–19]. However, there is now evidence that the hypersensitivity to unpleasant stimuli extends beyond the somatosensory system, which has led to the hypothesis that there is a generalized hypervigilance for sensory stimuli in FM [16, 20, 21]. A few studies have examined the sensitivity of FM patients in modalities other than pain and found perceptual amplification. FM patients have been shown to have decreased tolerance of unpleasant noise [20] and increased sensitivity to loud unpleasant auditory stimuli that parallels their increased pressure pain sensitivity [22]. Similarly, FM patients perceive unpleasant olfactory stimuli to be more intense and more unpleasant than do matched control subjects [23]. On the other hand, when pleasant odors were tested, FM patients and controls perceived the odors as equally intense, consistent with another evidence that the hypersensitivity across perceptual modalities may be confined to stimuli in the unpleasant range [24]. Nevertheless, for pleasant odors, although FM patients did not rate them as more intense, they did evaluate the pleasant odors as less pleasant than did control subjects. Further, a range of auditory stimuli were rated as more intense by FM patients than by controls, and auditory stimuli rated as mildly pleasant by healthy subjects were rated as somewhat unpleasant by FM patients [16]. The finding of hypersensitivity in multiple modalities of stimulation, particularly for unpleasant stimuli, suggests that the evoked pain sensitivity of FM may be related to an altered hedonic appreciation for sensory stimuli, rather than to peripheral tissue abnormalities. 

### 1.3. Other Phenomena Related to Altered Pain Perception

Other types of evidence from experimental pain studies in FM patients support the idea of a centrally mediated up-regulation of nociceptive activity in the CNS. A central pathophysiological process that appears to be disturbed in FM patients is the “windup” of central nociceptive processing of C-fibre input to the spinal cord, resulting in the perceptual phenomenon of temporal summation of pain. Windup of nociceptive activity is dependent on activation of the NMDA receptor complex in the spinal cord by input from C-nociceptors [25, 26]. Some FM patients show increased temporal summation of pain and increased aftersensations at the termination of noxious stimulation [27]. These enhanced responses could be related to one or more of several possible factors: (1) an ongoing peripheral source of input from C nociceptors other than the applied stimulus; (2) sensitized NMDA receptors on central nociceptive neurons; (3) abnormalities in descending modulation; (4) abnormal processing at supraspinal levels. Evidence of increased sensitivity in multiple sensory modalities suggests that ongoing C-nociceptor input cannot alone account for FM symptoms, indicating that there probably also are either sensitized NMDA receptors, abnormalities in modulatory systems in the brain, or abnormal sensory processing at spinal or supraspinal levels. Increased sensitivity has been demonstrated at the spinal level in FM [11]. Staud et al. [28] showed that an NMDA inhibitor reduced temporal summation in both healthy people and FM patients, suggesting that NMDA receptors probably are not sensitized in FM. On the other hand, experimental evidence shows that there are abnormalities in pain modulatory systems in FM patients that could account for altered temporal summation and other putative spinal effects.

### 1.4. Altered Pain Inhibition in FM Patients

For hundreds of years, clinicians have known that pain inhibits pain, a phenomenon termed “counterirritation.” More recently, a physiological basis of this phenomenon has been identified; the application of noxious stimulation activates an endogenous analgesic system involving supraspinal descending control of dorsal horn nociceptive activity. This system is termed “diffuse noxious inhibitory control” or DNIC and its physiological basis in the spinal cord has been studied extensively in anesthetized animals [29, 30]. Nevertheless, when competing noxious stimuli are presented in conscious humans, other systems that modulate pain, such as distraction, also are probably in effect, so that care must be taken in inferring that perceptual effects are due to DNIC. Accordingly, a group of interested researchers has suggested that the term “conditioned pain modulation” be used in humans studies to avoid the mechanistic implication [31]. Studies that have examined conditioned pain modulation in FM patients show that conditioning stimuli that produce an analgesic response to experimental pain stimuli in healthy control subjects fail to have an effect on FM patients [13, 32–34]. One of these studies controlled for the effects of distraction and habituation and found a similar lack of conditioned pain modulation in FM patients [33], suggesting the possibility that the DNIC system is in fact impaired in these individuals. Alternatively, DNIC and other descending inhibitory systems could be activated by the widespread pain of FM, and the failure to demonstrate DNIC in FM could represent a ceiling effect in which these activated systems cannot be further engaged by the experimental manipulations [8]. In addition, distraction can have a powerful pain-inhibiting effect [35–39], and some researchers have suggested that FM patients have altered attentional focusing, with a hypervigilance to unpleasant stimuli (see discussion above).

## 2. Other Symptoms of FM

### 2.1. Altered Cognitive Function in FM Patients

In addition to pain, many patients with fibromyalgia complain of problems with memory and concentration, often referred to as “fibrofog” [40–43]. This clinical symptom has received a large amount of experimental study, and studies using objective cognitive tests substantiate patients' subjective reports of cognitive dysfunctions, most commonly related to speed of information processing, attention, and memory [43–56]. The most robust deficits in tests of memory and attention have so far been observed in paradigms involving a prominent distraction from a competing source of information, wherein FM patients are less capable than healthy controls to retain new information when rehearsal is prevented by a distraction [49, 50, 57]. Milder deficits have been observed in memory free of distraction at encoding [43, 44, 48, 49, 51, 58, 59]. FM patients frequently display greater impairments in the ability to actively retrieve past episodic events in the absence of a cue (free recall) than on recognition tests, which serve to evaluate the retrieval of remembered information and are more resistant to the effects of impaired attention and concentration [43, 44, 48, 51]. It has thus been proposed that memory impairments in FM are more highly related to attentional factors that modulate the efficiency of memory functioning than to primary memory processes per se [48, 60, 61]. Thus, the inability to manage distraction seems to be a particular problem in fibromyalgia patients and is reflected in patients' reports of difficulty concentrating and dealing with complex, rapidly changing environments [61] and by memory tests showing performance decrements in the presence of distraction. Impaired cognitive performance is evident even after controlling for anxiety and depression and the influence of medications that might affect cognitive functioning [43, 50, 52, 58]. Another area of cognitive functioning that has been shown to be abnormal in FM is that of emotional decision making [62, 63]. A similar deficit has been shown in chronic back pain patients, suggesting that this is not unique to FM [64]. 

### 2.2. Sleep Disturbances in FM Patients

Many FM patients complain of unrefreshed sleep. Several laboratory studies using objective measures of sleep physiology such as EEG substantiate these reports by showing disordered sleep architecture in FM patients, including delayed onset to sleep, altered sleep stage dynamics, and reduced slow wave sleep (deep sleep) and rapid-eye movement (REM) sleep [65–68]. The intrusion of EEG frequencies characteristic of wakefulness (alpha waves) in the deep non-REM sleep (delta waves) seems to be a prominent feature of the nonrestorative sleep of FM patients [65, 69–71]. Further, patients with FM often have fragmented sleep resulting from periodic intrusions such as involuntary limb movements (restless legs), sleep apnea, and arousal disturbances [68, 72–74]. Although FM patients tend to report greater disturbances in sleep duration and quality than shown in laboratory studies, and their subjective reports correlate better with the severity of clinical symptoms [75], objectively measured sleep disturbances have been associated with pain and subjective daily sleepiness in several studies [67, 68, 71, 73]. 

## 3. Brain Changes That Could Underlie Symptoms

### 3.1. Neural Basis of Pain Amplification and Altered Pain Modulation

Functional brain imaging studies support psychophysical findings of increased pain perception in FM, in that there is an augmentation of sensory processing throughout pain-related brain regions [9, 76–81]. This is important, since laboratory findings of increased sensitivity could be interpreted as a reporting bias, rather than evidence of increased activation in pain pathways. The functional imaging studies have found that fibromyalgia patients show significantly more activity in response to pressure and thermal stimuli compared to controls in a number of brain regions. Increased activations were observed not only in limbic structures, but also in brain regions involved in sensory-discriminative processing, such as primary and secondary somatosensory cortices, which supports the view that neural responses to afferent signals are amplified in fibromyalgia. 

Although the increased pain-evoked brain activations corroborate patients' reports, the correlation between increased brain activity and increased pain perception does not explain how the afferent signal is amplified. As discussed above, there is psychophysical evidence of dysfunctions in pain modulation as well as pain perception. There is now much evidence that the activation of descending control circuitry is involved in pain modulation and that this circuitry includes parts of prefrontal, cingulate, and insular cortices [23, 36, 37, 82, 83]. A number of anatomical imaging studies in FM patients reveal decreased brain gray matter in these regions [84–90]. Although the cellular basis of decreased gray matter in FM patients is not known, it is possible that due to neuronal loss, decreased dendritic arborisation, or changes in glial activation, pain inhibitory systems do not work in FM patients as well as in healthy individuals. 

Consistent with the idea that pain modulatory systems may be disturbed in fibromyalgia are data showing that some FM patients have abnormalities in neurochemical systems involved in pain control, including the forebrain opioid and dopamine systems. A positron emission tomography (PET) competitive binding study using the D2/D3 receptor antagonist [^11^C] raclopride showed that striatal dopamine is released in response to painful muscle stimulation in healthy subjects, but not in FM patients [15, 91], which might partially explain the increased sensitivity of FM patients to the painful muscle stimulation. For the opioid system, investigators using PET found that FM patients had decreased binding potentials at rest for the exogenously administered *μ*-opioid receptor agonist carfentanil in several brain areas, including the ventral striatum, the anterior cingulate cortex, and the amygdala [92]. These areas are implicated in pain and its emotional modulation, and correspondingly, the binding potentials showed a negative relationship with the magnitude of affective pain scores relative to the sensory scores. Although results of this study do not tell us whether levels of endogenous opioids were increased or whether receptor availability was decreased, the findings support the notion that disturbances in the opioidergic system might be related to the increased pain sensitivity in fibromyalgia. For both dopamine and opioids, the ongoing widespread pain of FM could lead to a tonic activation within these systems and thus be a main factor in altering receptor availability and associated responsiveness to externally applied painful stimuli. 

### 3.2. Neural Basis of Cognitive Symptoms

It is well known that cognitive capabilities such as attention and memory functions decline continuously across the adult lifespan [93], which, together with findings of accelerated age-related decline of brain gray matter observed in FM patients [84], suggests that there may be a relationship between gray matter reductions in FM and cognitive deficits in these patients. Two recent studies have linked FM to impaired emotional decision making [62, 63]. Anatomical imaging studies have reported that FM patients have decreased gray matter in the medial prefrontal and insular cortices [84, 85, 89], areas implicated in emotional decision making [94–99]. Together, these data suggest a possible association between gray matter loss and emotional decision making in FM. One study has directly examined the relationship between performance on working memory tasks and gray matter in FM patients and found that an individual's performance was positively correlated with gray matter values in medial frontal and anterior cingulate cortices, thereby providing direct evidence for an association between altered working memory and gray matter morphology in fibromyalgia [51]. Both of these brain regions, together with lateral premotor cortex, lateral prefrontal cortex, frontal poles, and posterior parietal cortex, are areas known to be related to working memory processes [100–105]. In terms of the neurochemical abnormalities in FM discussed above, dopamine plays an important role for cognitive functioning. Multiple lines of evidence demonstrate the importance of mesocortical and striatal dopaminergic pathways in memory tasks, perceptual speed, and response inhibition (see [106] for review). Thus, there is an overlap between tasks in which fibromyalgia patients perform poorly and tasks that are related to dopamine functioning, suggesting that a dysfunctional dopamine system could contribute to the cognitive symptoms of fibromyalgia.

### 3.3. Neural Basis of Sleep Disturbances

While many studies have used EEG and related methods to show various aspects of disordered sleep physiology in FM patients, little is known about the neurobiology underlying these disturbances. Several neurotransmitters have been proposed to influence CNS hypersensitivity associated with sleep alterations. For example, inhibition of the CNS serotonin synthesis has been linked to insomnia and increased pain sensitivity [107]. Accordingly, in FM there is evidence for low serum and cerebrospinal fluid serotonin levels [108, 109]. Injecting amounts of substance P into the CNS of rats has been shown to reduce sleep efficiency, increasing latency to onset to sleep and provoking awakenings from sleep [110], and there is evidence for elevated cerebrospinal fluid levels of substance P in FM patients [111, 112].

### 3.4. What Do the Psychophysical, Cognitive, and Neuroimaging Studies Tell Us about the Neurobiology Underlying FM Symptoms?

The wealth of experimental evidence showing that FM patients are hypersensitive to painful stimuli, as well as unpleasant stimuli from other sensory modalities, in conjunction with functional brain imaging data showing increased stimulus-evoked activation throughout nociceptive pathways, shows that the defining symptom of FM—increased pain—is in fact real and not just a response bias of the patients. The finding that perception is increased in multiple modalities speaks against the hypothesis that FM pain is due to an upregulation of peripheral nociceptive processes. Further, psychophysical evidence that descending modulatory systems are altered in FM patients supports the opposing idea that FM symptoms are at least in part caused by alterations in CNS processing of the pain signal, including a dysregulation of pain modulatory systems. Nevertheless, the apparent dysregulation within these systems could be caused and/or perpetuated by a tonic activation related to the presence of ongoing widespread pain, so that the systems are saturated and cannot regulate further in response to external stimuli. 

Since similar descending control systems, including attentional and emotional regulatory circuitry, affect multiple sensory modalities [113–119], a dysfunction (or saturation) in these systems could lead to the hypersensitivity in multiple sensory modalities. FM patients show reduced habituation to nonpainful tactile stimuli and increased cortical response to intense auditory stimuli, both of which have been linked to deficient inhibition of incoming sensory stimuli [120, 121]. Also in support of the idea of a central dysregulation or saturation of pain modulation are changes in the opioid and dopamine neurotransmitter systems, both known to be involved in hedonic regulation [122]. 

Finally, the findings that FM patients not only perceive themselves to have altered memory and concentration (“fibrofog”), but also in fact perform poorly on multiple cognitive tests, even when depression is excluded as a contributing factor, suggest that there are alterations in brain function. The anatomical brain imaging studies that show reductions in gray matter in frontal regions important for cognitive function further indicate that this common symptom of FM is based on altered brain function. Together, the experimental evidence provides strong support for the idea that FM symptoms are related to dysfunctions in the central nervous system. The cause of these changes cannot be deduced from the available evidence, as it is correlational in nature. Did long-term ongoing pain cause the changes or did the changes cause the pain? Without a relevant animal model or long-term longitudinal studies, we cannot answer these questions. Nevertheless, we can at least say that fibromyalgia is real and that it is associated with multiple changes in the brain.
